# The Microscopic Origin of Residual Stress for Flat Self-Actuating Piezoelectric Cantilevers

**DOI:** 10.1007/s11671-010-9810-z

**Published:** 2010-09-30

**Authors:** Jeong Hoon Lee, Kyo Seon Hwang, Tae Song Kim

**Affiliations:** 1 Department of Electrical Engineering, Kwangwoon University, Seoul, 139-701, Korea; 2 Nano-Bio Research Center, Korea Institute of Science and Technology, Seoul, 136-791, Korea

**Keywords:** Nanomechanics, Residual stress, Piezoelectric, Cantilever, Biosensor

## Abstract

In this study, flat piezoelectric microcantilevers were fabricated under low-stress Pb(Zr_0.52_Ti_0.48_)O_3_ (PZT) film conditions. They were analyzed using the Raman spectrum and wafer curvature methods. Based on the residual stress analysis, we found that a thickness of 1 μm was critical, since stress relaxation starts to occur at greater thicknesses, due to surface roughening. The (111) preferred orientation started to decrease when the film thickness was greater than 1 μm. The d_33_ value was closely related to the stress relaxation associated with the preferred orientation changes. We examined the harmonic response at different PZT cantilever lengths and obtained a 9.4-μm tip displacement at 3 V_p-p_ at 1 kHz. These analyses can provide a platform for the reliable operation of piezoelectric microdevices, potentially nanodevice when one needs to have simultaneous control of the residual stress and the piezoelectric properties.

## Introduction

There is strong interest in the use of piezoelectric films applied to micro/nano-electro-mechanical systems (MEMS/NEMS) for sensing, actuating and energy-harvesting applications [1-3]. Among the piezoelectric materials, lead zirconate titanate (PZT) film, especially with a morphotropic phase boundary (MPB), is one of the most promising candidates for MEMS and NEMS applications, since it has high piezoelectric coefficients, high electromechanical coupling coefficients, and thermal stability.

Residual stress affects the piezoelectrical characteristics and reliability in films. More importantly, it plays key role in the reliability of the MEMS device structure. For example, in comparison with an electronic application, such as ferroelectric random access memories (Fe-RAMs), one has to maintain stress-free micro/nanostructure by controlling the residual stress. If one fails to control the residual stress, which can cause cracking, bending, and unintended electromechanical operation (i.e. frequency change), then one would fail to acquire a successful fabrication, and a reliable operation, and so the performance of the device would suffer.

In general, residual stress can be expressed as the sum of the intrinsic stress, the thermal stress, and the extrinsic stress. Previous reports have examined the effects of residual stress on the ferroelectric properties of Pb(Zr_*x*_Ti_1-*x*_)O_3_ (PZT) thin films [4,5]. One of the stress measurements, Raman spectroscopy, has been used to measure the residual microstress found in PbTiO_3_, PZT and Nd-modified PZT [6-8]. In these studies, a linear relationship was observed between the square of the Raman frequency and the residual stress.

Recently, we reported on the microstress found in Pb(Zr_0.52_Ti_0.48_)O_3_ (PZT) films using the Raman spectrum and the macrostress using the wafer curvature method. We showed that the piezoelectric response was related to the stress relaxation with a preferred orientation change [9]. We also presented the application of smart piezoelectric materials into cantilever-based biosensors.

To the best of our knowledge, although many efforts have been made, both in device application and in thin-film stress analysis, no one has yet reported on the relationship between the residual stress and the device fabrication to a low-stress flat microcantilever. Therefore, we present a study on a flat PZT microcantilever under a low-stress PZT film condition, which is analyzed by the Raman spectrum and the wafer curvature methods. We also show the harmonic response as well as the quasistatic tip deflection for a self-actuating microresonating device. These analyses can also provide a platform for the reliable operation of piezoelectric nanodevices such as nanobridge, nanocantilever, and nanoresonator in nanoelectromechanical systems (NEMS).

## The Experimental Procedure

### The Thin-Film Deposition and Analysis

We prepared PZT films using the chemical solution deposition (CSD) method based on 1-3 propanediol [9]. The films were prepared by spin coating the 0.5 M stock solution onto the Pt/Ti/SiO_2_/Si substrates and subsequently heating them at 400°C for 5 min and at 650°C for 10 min for each layer (interlayer annealing). We prepared the PZT films using interlayer annealing technique in order to prevent macro- and microcracking, which has been reported to be observed between the residual stress values of 117.21 and 198.14 MPa. It has been found that without interlayer annealing, cracks have been observed ranging from 0.6 μm up to 1.0 μm, whereas no cracks have been observed when using the interlayer annealing technique.

The microstress within the PZT of different film thicknesses was measured by Raman spectroscopy using a Raman spectrometer with backscattering geometries. The 514-nm line of an argon ion laser was used as the excitation source and calibrated using a silicon sample before measuring the PZT films. For the macrostress analysis, the radius of the curvature of the PZT films deposited onto the Pt/Ti/SiO_2_/Si (100 μm) substrates was measured using Tencor P1 equipment; the stress was then calculated according to the Stoney equation. The crystallinity and the phase identification of the film was analyzed using X-ray diffraction (XRD; Rigaku D/Max ШA) with Cu K α radiation (λ = 0.154 nm). The values of the preferred orientation parameter, α_hkl,_ can be extracted from the respective peak height ratios.

A commercial AFM (M5, Park Scientific Instruments (PSI) USA) and field emission scanning electron microscopy (FE-SEM; Hitachi S700) were used to examine the surface roughness, the grain size, the film thickness, and the device images. We examined the piezoelectric coefficient (d_33_) using the pneumatic loading method. For the piezoelectric characterization, a poling process was done under the field at 150 kV/cm for 15 min at 135°C on a hot plate.

### The Electromechanical Response of the Microcantilever

We measured the resonant frequency as well as the tip deflection using a heterodyne laser Doppler vibrometer (MLD211D, Neo ark Co. Japan). In order to measure the resonant frequency, an external 0.5 V_pp_ (peak to peak) AC sine wave with a superimposed dc voltage (0.25 V) was applied to the top electrode to vibrate the cantilever; the bottom electrode was grounded. The vibrating mechanical signal of a PZT microcantilever can be measured by a heterodyne laser Doppler vibrometer. The maximum cantilever deflection at the first resonant frequency can be detected. It was found that all of the frequency responses of the nanomechanical PZT microcantilever exhibit Lorentzian characteristics without severe electromechanical nonlinearity at the driving amplitude of 0.5 V_pp_. A tip deflection was obtained at 1 kHz for the quasistatic condition (out of resonant frequency).

## The Results and Discussions

**Figure 1:**
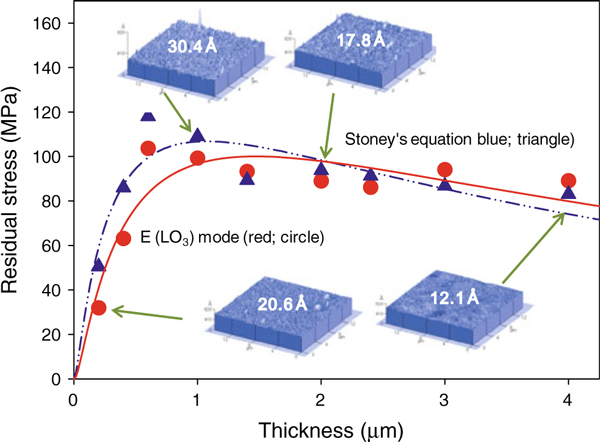
**The residual stress and surface roughness according to the PZT film thickness**. The microstress analysis was measured using the Raman spectra (*red circle*) taken from the frequencies of E(LO_3_) Raman modes, whereas the macrostress was calculated using the wafer curvature method (*blue triangle*). Taken from the AFM images, the rms roughness had a maximum value at a film thickness of 1 μm.

The residual stress and the surface roughness according to the PZT film thickness is shown in Figure 1. The residual stress of the films according to PZT film thicknesses was calculated from the Raman spectra and the Lydane–Sach–Teller relationship [10]. In the literature, macrostress has been defined as the sum of the stresses arising from the microstructural phenomena, such as ferroic domains, stacking faults, and dislocations, while microstress represents a homogeneous stress state distributed throughout the entire surface of the film and substrates [11]. In order to determine the microstress from the Raman spectra, the data was fitted using a linear background correction and the Gaussian peak shape. By observing the frequency change in the E(LO_3_) Raman modes, we could measure the microstress at different PZT film thicknesses (the red circle in Figure 1). Then, we fit all the data to the peak, log-normal, 3-parameter equation using the SigmaPlot^R^ software (Ver10, Systat Software Inc). The positive values in the microstress profile indicate that all of the microstresses were tensile stresses. To verify this microstress value, we calculated the macrostress at different film thicknesses using the wafer curvature method via the Stoney equation. Interestingly, the macrostress had a value close to that of the microstress obtained from the Raman spectra. From the results of the macrostress and microstress analyses, we clearly observed a rapid increase in the tensile stress up to a thickness of 1 μm. When the film thickness increased above 1 μm, the residual stress, analyzed both from macrostress and microstress, slowly decreased with an increase in the film thickness.

AFM topographic scans (the inset in Figure 1) of the PZT films showed that the root-mean-square (rms) roughness of the PZT films were 20.6, 30.4, 17.8, and 12.1 Å, when the film thickness was 0.2, 1.0, 2.0, and 4.0 μm, respectively. The residual stress was reported to be released due to stress–relaxation mechanisms, such as dislocation nucleation and multiplication, film cracking, and surface roughening [12,13]. The fact that the rms roughness had a maximum value at a 1 μm thickness could be related to these stress–relaxation mechanisms. The interlayer annealing technique used in this paper could keep the films from surpassing the critical stress levels, which can lead to cracking [14].

**Figure 2:**
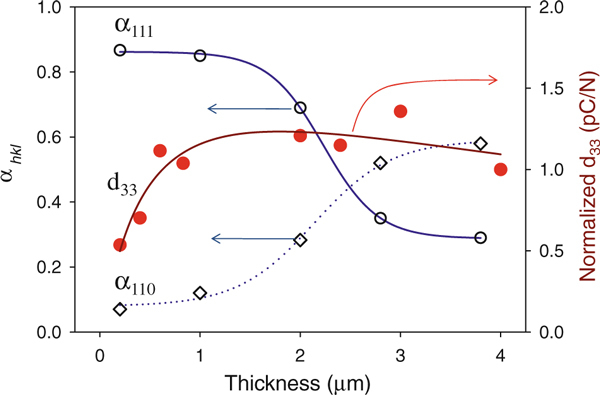
**a The preferred orientation (*α*_*hkl*_) from the X-ray diffraction analysis and b the normalized piezoelectric coefficient (d_33_) compared to the PZT film thickness**.

The values of the preferred orientation as well as normalized piezoelectric response are presented in Figure 2. The values of the preferred orientation parameter, α_hkl,_ are extracted through the respective peak height ratios of (100), (110), and (111) obtained from X-ray diffraction analysis. We observed a mixture of (100), (110), and (111) orientations in all of PZT films and calculated the preferred orientation (*α*_*hkl*_) values shown in Figure 2. As the film thickness increased, the preferred orientation of the PZT films changed from (111) to (110). When the film thickness became greater than 1 μm, *α*_*111*_ started to decrease while the *α*_*110*_ orientation started to increase. It was shown that 57% of the initial *α*_*111*_ changed to *α*_*110*_ when the thickness increased from 1 to 3 μm. Previous studies reported that the film orientation depends on the residual stress; the (111)-preferred increased as the tensile stress increased, the (100)-preferred increased as the compressive stress increased [15]. Therefore, it seems reasonable that the decrease in *α*_*111*_ when the thickness was greater than 1 μm was related to the relaxation of the tensile stress. It would be reasonable to infer that a build-up of residual stress up to a thickness of 1 μm could be released by the surface roughening effect and so the stresses at a thickness of 1 μm and above could be released by changes in the preferred orientation.

The piezoelectric coefficient (d_33_) at the different PZT film thicknesses is also shown in Figure 2. We measured the direct piezoelectric coefficient (d_33_) using a charge integration technique. We normalized the d_33_ value with a normalizing factor of 1 for the 1-μm-thick film, since it has been reported that a charge integration technique has a higher apparent piezoelectric coefficient due to the substrate bending effect [16]. The normalized d_33_ value was plotted using a peak, log-normal, 3-parameter equation. When this was done, it was clear that the d_33_ value rapidly increased up to a 1 μm thickness and started to decrease gradually as the thicknesses increased above 1 μm.

**Figure 3:**
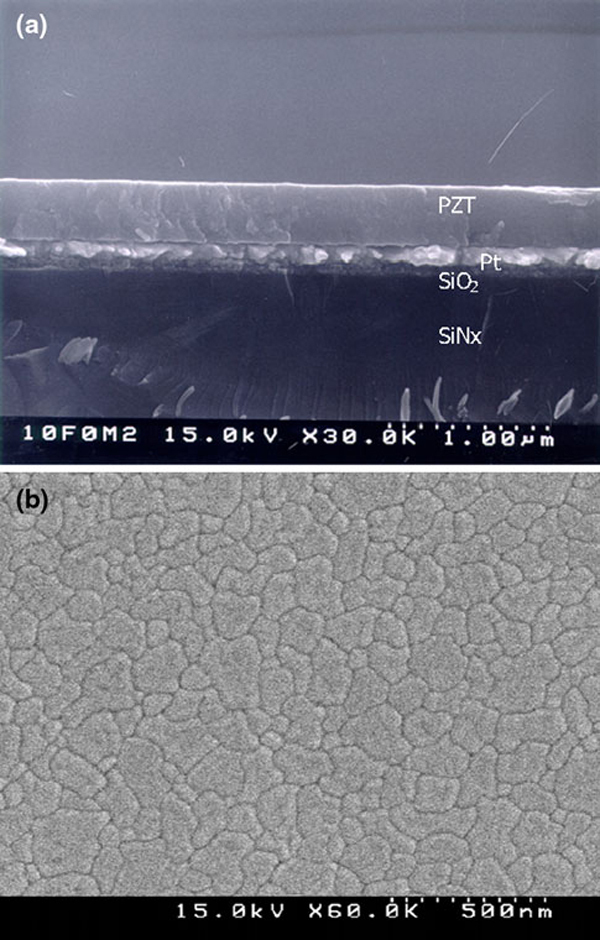
**The SEM images of a the cross-section and b the surface of the 0.4-μm-thick PZT film on the multilayered Pt/Ta/SiO_2_/SiN_x_/Si substrate**.

The fabrication of the piezoelectric MEMS device mainly consists of the multilayered film deposition and the etching process. We deposited PZT films with a 400 nm thickness, chosen in regard to the results of residual stress analysis. The residual stress at 400 nm thickness enabled films to not surpass the critical stress that could cause cantilever warping, bending, and cracking. In Figure 3, we show the cross-sectional and surface SEM images of the 400-nm-thick PZT films on the Pt (150 nm)/Ta (30 nm)/SiO_2_ (100 nm)/SiN_x_ (1.2 μm)/Si substrate. Based on the SEM images, we observe a good crystallized columnar microstructure and uniform grain size.

**Figure 4:**
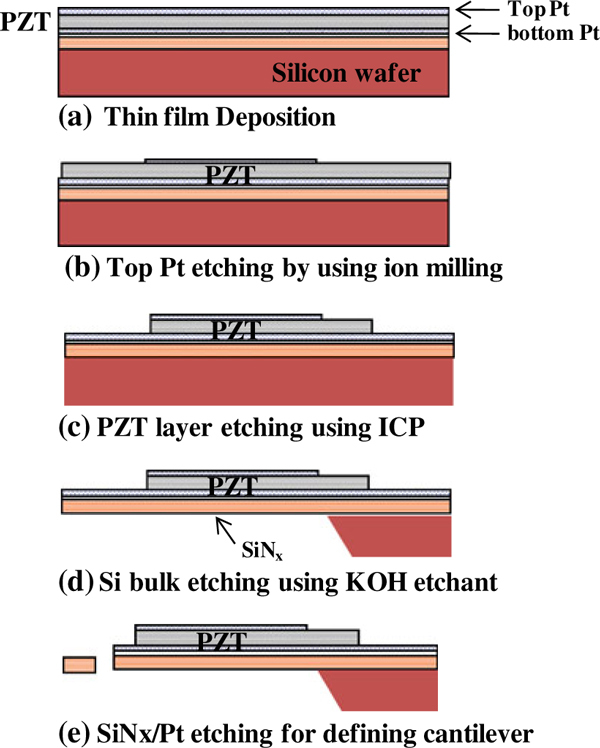
**The process flow chart for the formation of the piezoelectric microcantilevers**.

The nanomechanical PZT cantilevers were fabricated as shown in Figure 4. We started the microfabrication with a 100-mm-diameter p-doped Si (100) wafer. First, we deposited 1.2-μm-thick low-stress silicon nitride with a tensile stress at ~50 MPa (SiN_x_) deposited by LPCVD, followed by a 100-nm SiO_2_ layer. The bottom electrode was then prepared by sputtering a thin Ta adhesion layer (30 nm) and a Pt layer (150 nm). The PZT films were deposited at the thickness of 400 nm. For the MFM (metal–ferroelectric–metal) capacitor structure, a Pt layer (100 nm) was deposited as the top electrode by DC sputtering.

The top Pt electrode was formed using standard photolithography and ion milling. The PZT layer etching was then carried out using inductively coupled plasma (ICP) etching. After the lithography process, the back of the SiN_x_ was etched using reactive ion etching (RIE), followed by bulk silicon etching using a KOH silicon etchant. Finally, the top SiN_x_ as well as the bottom Pt layers were etched using RIE to define the cantilever.

**Figure 5:**
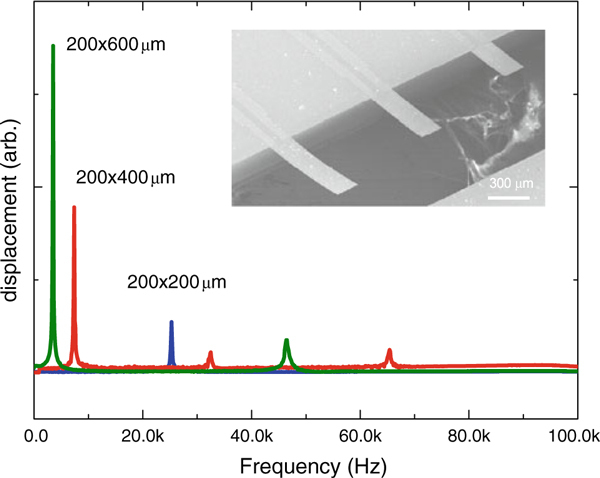
**The harmonic response of the three different length self-actuating piezoelectric cantilevers**. The inset SEM photograph shows flat cantilever arrays. The length of the cantilevers were 200, 400, and 600 μm with a 2.05-μm thickness.

The SEM photographs of the PZT microcantilever arrays (the inset images in Figure 5) show flat PZT cantilever arrays with self-actuating and sensing functions. The lengths of cantilevers were 200, 400, and 600 μm, with a constant width of 200 μm. The total thickness of the PZT cantilevers was 2.05 μm, and the residual stress of the 400-nm-thick PZT layer taken from the residual stress analysis (Figure 1) was 40 ± 10 MPa. Actually, if one fails to control the residual stress, the residual film stress can cause device bending, as shown in previous reports [17]. As seen from the residual stress analysis, we acquired low-stress flat PZT cantilevers, which are shown in the SEM photographs.

We examined the harmonic response at different PZT cantilever lengths (Figure 5). The resonant frequency of a piezoelectric unimorph cantilever can be written as:

(1) $$f=\frac{\upsilon_{n}^{2}}{2\pi }\;\frac{1}{L^{2}}\sqrt{\frac{\left(\bar{EI}\right)}{\left(\rho_{np}h_{np}+\rho_{p}h_{p}\right)}}$$

where $\bar{EI}$ can be written as:

(2) $$\frac{1}{\left(\bar{EI}\right)}=\frac{12}{w}\;\frac{E_{np}h_{np}+E_{p}h_{np}}{E_{np}^{2}h_{np}^{4}+E_{p}^{2}h_{p}^{4}+E_{np}E_{p}h_{np}h_{p}(1h_{np}^{2}+6h_{np}h_{p}+4h_{p}^{2})}$$

In these equations, a piezoelectric unimorph cantilever has Young's modulus *E*, the moment of inertia *I*, the thickness *h*, the width *w*, and the length *L*. $\upsilon_{\text{n}}^{2}$ is a dimensionless *n*th-mode eigen value, and '*np*' and '*p*' denote the elastic nonpiezoelectric layers composed of SiN_*x*_, SiO_2_, Pt with the thicknesses of 1.2 μm, 100 nm, and 250 nm, respectively, and the piezoelectric thin film with a thickness of 400 nm, respectively. The parameters used for the theoretical calculation of the PZT unimorph cantilever can be found elsewhere [18-20]. In the case of the resonant frequency of an unclamped cantilever, the eigen value of υ_*n*_ is 1.87, 4.69, and 7.85 for the 1st, 2nd, and 3rd harmonics, respectively. Taken from the parameters of the PZT and SiN_*x*_/SiO_2_/Pt, the theoretical 1st resonant frequency with the dimensions of 200 × 600 μm, 200 × 400 μm, and 200 × 200 μm are 4.85, 10.9, and 43.7 kHz, whereas the experimental measured results are 3.5, 7.38, and 25.3 kHz, respectively. The reason for the down-shift of the measured resonant frequency compared to the theoretical values could be from the microfabrication. It is reasonable to infer that the cantilever could be over etched along the cantilever length during the KOH Si wet-etching process. If one considers a 60-μm undercut of the cantilever during the KOH etching, the theoretical value corresponds exactly to the experimental one.

**Figure 6:**
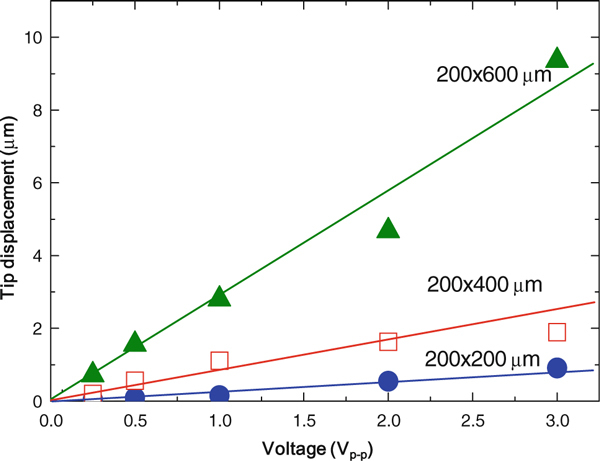
**The applied voltage dependence of the tip displacement of the PZT cantilever beams with the three different lengths**. The tip deflection was measured at 1 kHz (quasistatic).

Figure 6 shows the tip displacements versus the applied voltage (V_p-p_). Although the displacements versus the frequency shown in Figure 5 represent a good linearity with *L*^2^ according to the theoretical equation, their displacements at the resonant frequency exhibit very large values that the vibrometer could not measure. For this reason, we operated the PZT cantilever at 1 kHz. It was clear that the tip displacement shows a good linearity with the *L*^2^ and the applied voltage (V) according to the following theoretical equation:

(3) $$\delta =\frac{3d_{31}S_{p}S_{np}h_{np}(h_{np}+h_{p})L^{2}V}{{\left(S_{np}\right)}^{2}{\left(h_{p}\right)}^{4}+4S_{np}S_{p}{\left(h_{np}\right)}^{2}h_{p}+4S_{np}S_{p}h_{p}{\left(h_{np}\right)}^{3}+{\left(S_{np}\right)}^{2}{\left(h_{np}\right)}^{4}}$$

where *d*_*31*_ is the piezoelectric coefficient, *S*_*p*_ and *S*_*np*_ are the elastic compliance of the piezoelectric and non-piezoelectric materials of the cantilever, respectively. *L* is the cantilever length and *V* is the applied voltage. The tip deflection at 1 V_p-p_, from three different cantilever lengths (*L*) at 200, 400, and 600 μm, was measured at 0.15, 1.1, and 2.8 μm, respectively. From the results, we acquired a 9.4-μm tip displacement at 3 V_p-p_ at 1 kHz.

## Conclusions

We present a flat PZT microcantilever under low-stress PZT film conditions, which were analyzed by using the Raman spectrum and wafer curvature methods. It was found that the film thickness that had the maximum rms roughness via surface roughening provides the required thickness information needed for fabricating the low-stress PZT cantilever. From the harmonic response as well as the quasistatic tip deflection, we acquired a 9.4-μm tip displacement at 3 V_p-p_ at a 1 kHz quasistatic condition. In the current analysis, the dimension of device is still micrometer scale with a submicron thickness; however, we actually use same analyzing platform to measure the residual stress as well as the electromechanical properties in a nanoscale device, suggesting that these analyses will be possible to provide a platform for the reliable operation of piezoelectric nanodevices, such as biosensors and energy-harvesting applications. In addition, stress-free flat cantilever incorporating multilayered piezoelectric materials could offer a common platform for label-free quantitative analysis of protein–protein binding, DNA hybridization and DNA–protein interactions as a nanomechanical sensor that could measure steady-state deflection with the tens of nanometer scale.
